# Unveiling the hidden risks: 90-day mortality and complications in older adults with proximal femur fractures

**DOI:** 10.1007/s40520-025-03134-0

**Published:** 2025-07-19

**Authors:** Clemens Roitzsch, Franziska Beyer, Klaus-Dieter Schaser, Roman Riedel, Marcel Mäder, Anne Postler

**Affiliations:** University Center of Orthopedics, Trauma and Plastic Surgery, University Hospital Carl Gustav Carus Dresden, TU Dresden, University of Technology, Fetscherst. 74, 01307 Dresden, Germany

**Keywords:** Geriatric, Proximal femur fracture, Mortality, Risk factors

## Abstract

**Background:**

Proximal femur fractures (PFF) are the most common fracture type worldwide, particularly in geriatric patients. Despite advancement in treatment, mortality rates in this patient group remain high.

**Aims:**

The aim of this study was to analyze potential factors, influencing the 90-day mortality rate and complication rate after PFF surgery in a tertiary care hospital.

**Methods:**

Between 2019 and 2022, all patients aged 65 and older with PFF and a positive screening as a geriatric patient were included in this prospective, single-center study. Data collected included gender, age, BMI, fracture type, type and timing of surgery, comorbidities, medication, mobilization and blood loss as well as complications and 90-day mortality.

**Results:**

Out of 1217 analyzed geriatric patients, 61% underwent surgery within 24 h. The complication rate was 21.7% with 3% surgical and 20.2% non-surgical complications. Median age was 87 years and 68% were female, while mean Charlson Comorbidity Index was 2.6 points and 84% had ASA 3 or 4. 240 patients (19.7%) died within 90 days after surgery. Higher mortality rates were associated with male gender (OR 1.341), higher age (OR 1.093), lower BMI (OR 0.946), higher Charlson Comorbidity Index (OR 1.202), non-surgical complications (OR 2.541) and if mobilization was impossible (OR 10.013). A delay of surgery beyond 24 h after admission was associated with the development of wound infections (*p* = 0.022) and internal medicine-related complications (*p* = 0.002).

**Discussion:**

While no correlation was found between surgery timing and 90 days mortality, delays in surgery were associated with adverse effects, including wound infections and internal medicine complications.

**Conclusion:**

The mortality rates of patients suffering from PFF maintain high and are mainly influenced by non-modifiable patient-related factors.

**Clinical trials registration:**

German Registry for Clinical Trials, ID: DRKS00034048, date of registration: 10.02.2025. Retrospectively registered.

## Introduction

Medical advancements and therapeutic interventions have significantly augmented global life expectancy, rising from 67 to 73 years over the past two decades [1]. Consequently, the percentage of people older than 65 years in Germany will increase from 20% in 2008 to 34% in 2060 [2]. With increasing life expectancy and a growing proportion of elderly individuals in the population, the incidence of osteoporotic fractures, especially proximal femur fractures (PFF), is expected to rise markedly. The global incidence of PFF is anticipated to escalate from 1.26 million in 1990 to 4.5 million by 2050 [3]. In Germany alone, over 155.000 patients were treated for PFF in 2019, rendering it the most prevalent fracture type. In comparison with 2009, this constitutes a notable increase of 24% in intertrochanteric fractures and 23% in femoral neck fractures [4].

Geriatric patients are often suffering from multiple comorbidities. Sustaining trauma, particularly PFF, can precipitate devastating setback and lead to significant reductions in quality of life and mobility [5]. Following PFF, patients frequently encounter challenges in regaining ambulation independence or mastering activities of daily living [6]. Despite improving the treatment of PFF, mortality rates in this patient cohort remain high with up to 36% one-year mortality [7].

Therefore, focus has been on improving therapy and management for geriatric patients with PFF. Several studies have shown that delayed surgery after 24–48 h is associated with increased mortality and complications [8–12]. Therefore, most national guidelines recommend to perform surgery within this time period [13]. However, the optimal timing for surgery remains a subject of ongoing debate, particularly in patients with severe preexisting conditions [13].

Since 2021, the resolution of the German Federal Joint Committee (Gemeinsamer Bundesausschuss der Krankenkassen und Ärzte, GBA) mandates surgical intervention to be performed within 24 h of admission and consequent implementation of interdisciplinary treatment.

The aim of this study was to analyze potential cofactors, influencing the mortality rate within 90 days after surgery as well as the rate of complications in PFF in a tertiary care hospital, giving special consideration to the effect of implementing the GBA-requirements of 24 h between hospital admission and surgery. Ethical approval for this study was granted by the Ethics Committee of the University Medicine Carl Gustav Carus, TU Dresden in 2021 (BO-EK 488102021).

## Materials and methods

**Fig. 1:**
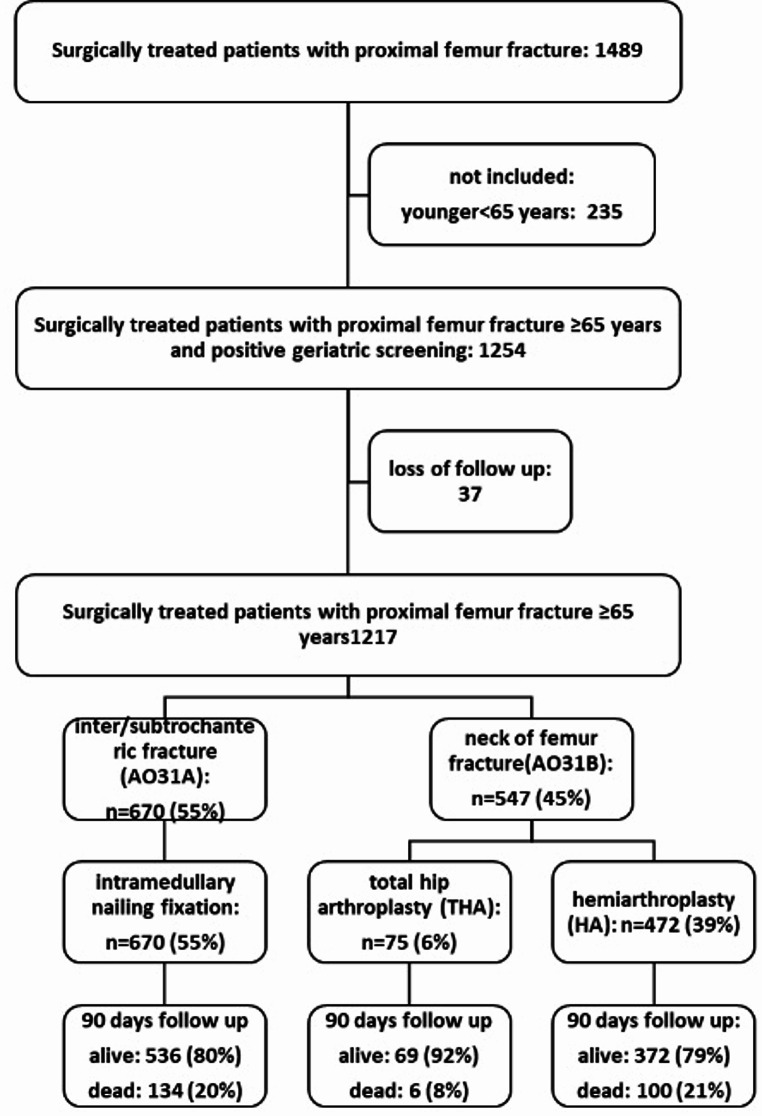
Flowchart

In this single center prospective cohort study, all geriatric patients aged 65 years and older with PFF who were treated surgically between 01/2019 and 12/2022 were identified (Fig. 1).

Geriatric patients were identified through positive screening in the emergency department based on criteria such as care level, nursing home residency, age 85 years or older, or dementia [14].

Inclusion criteria were patients aged 65 years and older with a PFF and positive geriatric screening.

Exclusion criteria were conservatively treated fractures, isolated greater trochanteric fractures, periprosthetic fractures as well as revision surgery in previously fixed fractures. Surgery was performed by trauma surgeons at our academic level 1 trauma center in Germany.

Collected data included demographic information (gender, age, BMI), comorbidities (American Society of Anesthesiologists classification, ASA [15], and Charlson Comorbidity Index, CCI [16]), medication and fracture morphology. Fractures were classified as femoral neck fractures, intertrochanteric and subtrochanteric fractures. Surgical interventions were selected based on fracture type and morphology. For femoral neck fractures (AO31B), patients’ age, preoperative activity, mobility, comorbidities and pain due to preexisting osteoarthritis of the hip determined whether total hip arthroplasty (THA) or hemiarthroplasty (HA) was performed. Intertrochanteric fractures (AO31A) were treated using cephalomedullary interlocking nails (PFN-A, Depuy Synthes), optional -if required- combined with cement augmentation of the femoral neck blade. Fixation of subtrochanteric fractures also involved intramedullary nailing using the PFN-A most often, additionally combined with cable cerclage following minimal-invasive open reduction of the fracture. Surgery was performed either in spinal, general or regional anesthesia depending on patients’ comorbidities and previously taken blood thinners. We recorded duration to and time of surgery, as well as intraoperative blood loss, postoperative mobilization and all inhouse-complications without a lethal outcome. The documentation of complications was based on a standardized complication form used in the department, which is to be completed upon patient discharge. All surgical complications, the need for reoperations, and non-surgical complications such as delirium, pneumonia, or acute kidney failure were recorded.

In-house and 90-days mortality were analyzed. 90-days mortality was registered by hospital information system and postal or telephone requests with patients and their relatives. Patients were subsequently divided into a survival and death group. No formal sample size calculation was performed, as all eligible geriatric patients treated between 2019 and 2022 were included. However, we followed the commonly used rule of thumb suggesting that for binary outcomes, there should be approximately 10–20 events per predictor variable [17].

Statistical analysis between groups was performed using the Mann-Whitney U test for continuous values not normally distributed and the chi-square test for categorical values. A multivariable binary logistic regression model was employed to identify factors associated with mortality after 90 days, with only those factors exhibiting a significance level below 0.2 in the univariate analysis included in the final model, using a backward elimination strategy. Odds Ratios (OR) with 95% Confidence Intervals (CIs) were estimated. Cumulative incidences of death up to 90 days post-operation were calculated using the Kaplan-Meier estimator. A p-value threshold of 0.05 was considered statistically significant. To assess the models fit, we calculated the Nagelkerke R². All data analyses were performed using SPSS (release 28.0 for Windows).

## Results

Between January 2019 and December 2022, 1489 patients 65 years and older suffering from PFF were treated. Of these, 1217 patients were identified as geriatric by the geriatric screening [14]. 37 patients (mean age 86, male/female 11/26) had to be excluded from analysis because the vital status after 90 days could not be determined. The mean age was 86 (range 65–105) and 68% (*n* = 826) were female. 84% (*n* = 1020) of patients had ASA 3 or 4 and median CCI was 2 (range 0–12). Table one provides characteristics of the study population.

**Table 1:** Patients characteristics, [interquartile range] ( †Chi-squared test; ‡Mann-Whitney U test) bmi = body mass index, asa = american society of anesthesiologists, cci = charlson comorbidy index, doac = direct oral anticoagulants

	Overall *N* = 1217		Alive at 90 days *N* = 977		Dead at 90 days *N* = 240		*p*-value
Median age. yrs	86.6	(7.3) [81.7; 91.0]	85.9	(7.1) [81.0; 89.8]	90.3	(7.3) [85.4; 93.4]	**< 0.001 ‡**
male gender. n	391	(32.1%)	299	(30.6%)	92	(38.3%)	**0.033 ‡**
Median BMI kg/m²	23.7	(4.3) [21.2.; 26.7]	23.9	(4.3) [21.5; 27]	23.3	(4.0) [20.8; 25.2]	**0.004 ‡**
**Comorbidities**							
Congestive heart failure	384	(31.6%)	278	(28.5%)	106	(44.2%)	**< 0.001 †**
Chronic renal insufficiency	659	(54.1%)	496	(50.8%)	163	(67.9%)	**< 0.001 †**
Dementia	473	(38.9%)	341	(34.9%)	132	(55.0%)	**< 0.001 †**
Diabetes mellitus	326	(26.8%)	260	(26.6%)	66	(27.5%)	0.759
Chronic pulmonary disease	175	(14.4%)	143	(14.6)	32	(13.3%)	0.736
Cerebrovascular disease	259	(21.3%)	197	(29.2%)	62	(25.8%)	0.055
Coronary heart disease	237	(19.5%)	177	(18.1%)	60	(25.0%)	**0.016 †**
Median CCI	2.6	(2.1)	2.4	(2)	3.3	(2.2)	**< 0.001 ‡**
**Antithrombotics**							
No Antithrombotics	830	(68.2%)	694	(71.0%)	136	(56.7%)	**< 0.001 †**
DOAC	341	(28.2%)	253	(25.9%)	88	(36.7%)	**< 0.001 †**
Thrombocyte aggregation inhibitor	16	(1.3%)	11	(1.1%)	5	(2.1%)	0.237
Vitamin K antagonist	29	(2.4%)	20	(2.0%)	9	(3.8%)	0.116
**ASA grade**							
1 to 2	197	(16.2%)	182	(18.6%)	15	(6.3%)	**< 0.001 †**
3 to 4	1020	(83.8%)	795	(81.4%)	225	(93.8%)	
Median preop Hb-Level	7.6	(1.1) [6.8; 8.3]	7.6	(1.1) [7.0; 8.4]	7.3	(1.2) [6.4; 8.1]	**< 0.001 †**

A total of 55% (*n* = 670) patients underwent surgical treatment using an intramedullary nail, while 39% (*n* = 472) and 6% (*n* = 75) received HA and THA, respectively. Median time until surgery was 22 h (range from 0.5 h to 24 days), Three patients had their surgery delayed for more than 10 days after admission, as they initially declined the operation.

739 (61%) Patients underwent surgery within 24 h, 403 (33%) between 24 and 48 h and 75 (6%) patients after 48 h after hospital admission (see table two). The most frequent reasons for postponing surgery were the intake of any kind of anticoagulation and/or the absolute need for preoperative optimization of patient’s health condition.

**Table 2:** Perioperative parameter, [interquartile range] ( †Chi-squared test; ‡Mann-Whitney U test)

	Overall *N* = 1217		Alive at 90 days *N* = 977		Dead at 90 days *N* = 240		*p*-value
**Surgical procedure**							
Total Hip Arthroplasty	75	(6.2%)	69	(7.1%)	6	(2.5%)	**0.028 †**
Hemiarthroplasty	472	(38.8%)	372	(38.1%)	100	(41.7%)	
Intramedullary nailing fixation	670	(55.1%)	536	(54.9)	134	(55.8%)	
**Anesthesia**							
General	516	(42.4%)	402	(41.1%)	114	(47.5%)	0.198
Spinal	638	(52.4%)	524	(53.6%)	114	(47.5%)	
Regional	63	(5.2%)	51	(5.2%)	12	(5%)	
**Mean time to surgery**	22.0	38.2	21.5	41.9	23.1	16.0	**0.02 †**
< 24 h	739	(60.7%)	605	(61.9%)	134	(55.8%)	0.219
24–48 h	403	(33.1%)	313	(32.0%)	90	(37.5%)	
> 48 h	75	(6.2%)	59	(6.0%)	16	(6.7%)	
Median duration of surgery. min	65.0	29.7 [43; 82]	65.0	29.4 [43; 82]	61.5	31.1 [46; 79]	0.258
Median duration of anesthesia. min	151.0	45.2 [125; 181]	150.0	43.4 [123; 180]	157.0	51.1 [130; 189]	**0.022 †**
Median nights at ICU	0.0	2.6 [0;1]	0.0	1.3 [0;0]	1.0	4.9 [0; 2]	**< 0.001 ‡**
Median Hb 3 d postoperative. mmol/l	5.6	0.9 [5.0; 6.3]	5.6	0.9 [5.0; 6.3]	5.6	0.9 [5.0; 6.1]	0.214
Median blood loss. l	1.3	1.0 [0.9; 2.0]	1.3	0.9 [0.9; 1.9]	1.5	1.2 [0.9; 2.2]	**0.014 †**
Median erythrocyte concentrates total	0.0	2.0 [0; 2]	0.0	1.7 [0;2]	1.0	2.7 [0;3]	**< 0.001 †**
**Mobilisation postop**							
Full weight bearing	1066	(87.6%)	881	(90.2%)	185	(77.1%)	
Partial weight bearing	115	(9.4%)	91	(9.3%)	24	(10%)	**< 0.001 ‡**
No mobilisation possible	35	(2.9%)	5	(0.5%)	30	(12.5%)	
Surgical complications	37	(3.0%)	27	(2.8%)	10	(4.2%)	0.257
Nonsurgical complications	246	(20.2%)	144	(14.7%)	102	(42.5%)	**< 0.001 ‡**

Overall, 229 patients with non-lethal inhouse-complications were recorded (19%). Among these, the rate of surgical complications was only 3% (38 patients with 39 complications) and revision was required in 21 cases (54%). The majority of patients experienced non-surgical complications (212 patients with 247 complications, 17%), such as delirium, pneumonia or acute kidney dysfunction. The difference between surgical and non-surgical complications was significant (*p* < 0.001). Tables three provides an overview of surgical and non-surgical complication.

**Table 3:** Overview of surgical complications (38 patients with 39 complications were recorded) and non-surgical complication (212 patients with 247 complications were recorded)

Surgical complications	39 (3% of 1217 patients)	Non-surgical complications	247 (17% of 1217 patients)
Dislocation	3 (8%)	*Delirium*	70 (28.3%)
Nerve damage	7 (18%)	*Pneumonia*	43(17.4%)
Intraoperative fracture	5 (13%)	*Thrombosis/pulmonary embolism*	22 (8.9%)
Impaired wound heeling	2 (5%)	*Acute kidney dysfunction*	38 (15.3%)
Osteosynthesis failure	5 (13%)	*Decompensated heart failure/myocardial infarction*	21 (8.5%)
Wound infection	11 (28%)	*Electrolyte imbalance*	16 (6.5%)
Postoperative Hematoma	6 (15%)	*Others*	37 (15%)
Revision required	21 (54%)		

The mortality rate at 90 days post-surgery amounted to 19.7%, corresponding to 240 patients. Additionally, an in-house mortality rate of 5% was observed, involving 61 patients.

Logistic regression models were performed to identify factors associated with 90-days mortality in patients with PFF after surgical treatment. The results are presented in table four. The final model showed a Nagelkerke R² of 0.336, indicating a moderate to good level of explanatory power. No associations were found between modifiable surgical parameters such as time to surgery, duration of surgery nor time or type of anesthesia. The type of fracture (femoral neck or intertrochanteric) showed no significant link to 90-days mortality (*p* = 0.786). Significant associations were discovered between 90-days mortality and male gender, higher age, lower BMI, higher CCI, preexisting medication with blood thinners, postoperative non-surgical complications and if mobilization was impossible.

Although no connection was found between the delay of surgery and 90 days mortality, a delay of surgery of more than 24 h after admission was associated with an increased risk of wound infections (*p* = 0.022) and internal medicine-related complications (*p* = 0.002).

**Table 4:** Results of multivariance analysis, risk factors for increased 90d mortality following PFF, bmi = body mass index, asa = american society of anesthesiologists, cci = charlson comorbidy index

Variable	Reference	OR	95% CI	*p*-value
Gender	Men	0.654	(0.442; 0.966)	**0.033**
Age	Per 1 year	1.102	(1.070; 1.135)	**< 0.001**
BMI	Per 1 kg/m²	0.938	(0.897; 0.980)	**0.004**
ASA	1/2	1.491	(0.791; 2.811)	0.217
CCI	Per 1	1.201	(1.106; 1.305)	**< 0.001**
Time to surgery	Per hour	0.994	(0.985; 1.003)	0.186
duration of anesthesia	Per min	1.003	(0.999; 1.007)	0.163
**Surgical procedure**				
Hemiarthroplasty	THA	1.276	(0.422; 3.860)	0.666
Intramedullary nailing fixation	THA	1.119	(0.363; 3.446)	0.845
Nights at ICU	Per night	1.190	(1.097; 1.291)	**< 0.001**
Antithrombotics	No	1.470	(0.989; 2.187)	0.057
Preoperative Hb	Per 1 mmol/l	0.878	(0.740; 1.042)	0.135
Blood loss	Per 1 L	0.886	(0.691; 1.135)	0.339
Erythrocyte concentrate	Per 1	1.082	(0.963; 1.215)	0.186
Nonsurgical complications	No	2.437	(1.646; 3.608)	**< 0.001**
**Mobilization**				
Full weight bearing	Partial	0.867	(0.453; 1.658)	0.666
Mobilization possible	Mobilization impossible	11.067	(3.625; 33.791)	**< 0.001**

## Discussion

This study analyzed mortality rates and risk factors in geriatric patients with PFF treated in a tertiary care hospital in Germany between 2019 and 2022. We observed a 90 days mortality rate of 19.7% and an in-house mortality rate of 5%. Several risk factors, including male gender, higher age, lower BMI, higher CCI, postoperative non-surgical complications, and inability to mobilize, were identified.

Compared to the literature, 90-day mortality rates vary between 8 and 22%. Studies with lower mortality rates typically include younger patients aged 50 years or older without geriatric screening and tend to report lower mean ASA scores than those in the present cohort [18–21]. Another important distinction lies in the median age of the patients. Age has shown to be one of the main risk factors for an elevated 90 days mortality. In our study, the median age of the patients, who died within 90 days after the operation, was over 90 years. These findings align with a meta-analysis from 2012, which identified patient-related factors as strong predictors of early postoperative mortality after PFF [22]. A recently published study by Pehkonen et al. showed a 90-days mortality rate of 9.9% for women and 16.1% for men. In-line with these results, our study also discovered male gender as a risk factor for increased mortality after 90 days. However, the reported mortality is notably lower than in our study, although their patients were younger on average [23].

The association of age and mortality compared to an increasing life expectancy [1] would suggest an increasing mortality rate over the years. Interestingly, mortality rates did not significantly change over the last decades [24], which could be explained with an increased focus on this vulnerable patient cohort and an optimized treatment within the last years. Especially interdisciplinary orthogeriatric treatment has shown to significantly reduce mortality [25]. However, some authors question whether a plateau in early postoperative mortality has been reached [26]. The most important risk factors concerning 90 days mortality are unmodifiable and patient-related factors like age, gender, comorbidities and BMI, as shown in this study. Therefore, modifiable perioperative factors concerning 90-days mortality have attracted major interest and attention within the last decades. Regarding the timing of surgery, recommendations in the literature vary, with suggested time frames ranging from 6 to 72 h [27–30]. However, since 2021 the German government and Federal Joint Committee, GBA, mandates surgery for PFF within 24 h [31] In this study 61% of the patients underwent surgery within 24 h, 33% between 24 and 48 h and 6% after 48 h. A significant association between the timing of surgery and 90-day mortality was observed in our univariate analysis; however, this correlation did not persist in multivariate analysis. Additionally, our results show that a delay of surgery of more than 24 h is associated with a higher incidence of wound infections and internal medicine-related complications, underscoring the importance of prompt surgical intervention. However, other studies have shown an association between mortality after 30 or 90 days and a delayed surgery of more than 24 h [9, 32]. Notably, Beaupre et al. demonstrated that patients older than 85 years were more likely to be negatively affected by increasing time to surgery. However, they did not give reasons for the postponement. Delays exceeding 48 h in our study were typically due to the need to optimize anticoagulation therapy or medical comorbidities preoperatively. These findings are supported by studies indicating that delays because of optimizing critically ill patients did not result in higher mortality [33, 34].

The overall complications rate in our cohort was 19%, with 3% of complications being surgical and 17% related to internal medicine, which is consistent with the literature [18, 35]. The most frequent complications were delirium, pneumonia and acute kidney failure, which were predominantly internal medicine-related. These findings underscore the importance of close interdisciplinary cooperation, particularly with orthogeriatric teams, to therapeutically address decisive comorbidities and reduce the risk of adverse outcomes [21].

Regarding anesthesia, no significant differences in 90-day mortality were observed between patients receiving spinal versus general anesthesia. However, a recent study using data from the American College of Surgeons National Surgical Quality Improvement Program, which included over 40,000 patients, reported a higher risk of 30-day mortality and postoperative complications (such as stroke, myocardial infarction, and acute kidney failure) with general anesthesia [36]. These findings suggest potential advantages of spinal anesthesia, particularly for patients with higher ASA scores, and should be considered during preoperative planning.

Postoperative mobilization should be prioritized as it is a major risk factor for increased 90-day mortality, with an odds ratio (OR) of 11 for patients who were unable to mobilize due to preexisting comorbidities or inability to walk prior to the fall. In our cohort, almost 90% of patients were able to perform full weight-bearing mobilization postoperatively. However, in 9% of patients, due to complex, comminuted fracture types and severe osteoporosis, the fracture fixation was not sufficiently stable in order to allow partial weight-bearing from the very beginning. Studies have shown that elderly patients often struggle to adhere to partial weight-bearing instructions, necessitating the use of wheelchairs for these individuals [37].

The following strengths and limitations need to be noted. While this was a prospective study, it was conducted at a single German level-1 center and therefore potentially open for selection bias. Our findings may not be generalizable to all hospital settings, as hospital size and level does not have an impact on survival but seems to affect time to surgery and complication rate [38]. Besides, residual confounding cannot be ruled out, despite multivariable adjustment. Additionally, we did not collect data on preoperative mobilization status, which could have a significant impact on postoperative recovery and mobility outcomes. Furthermore, we recorded only in-house complications. Complications occurring after discharge or requiring admission to another hospital due to complications remained unknown.

## Conclusion

Mortality rates in patients with PFF remain high and are primarily influenced by patient-related non-modifiable factors such as age, gender, comorbidities, and BMI. Surgical complications were relatively rare, while most complications were internal medicine related which had a significant impact on 90-day mortality. No significant correlation was found between the timing of surgery and 90-day mortality. However, delays beyond 24 h were associated with an increased risk of wound infections and internal medicine complications, which in turn significantly and adversely affect survival. To improve patients’ outcomes and reduce mortality for this vulnerable patient population, physicians should prioritize the following measures in order to address the causative prognostic factors for improved survival found in the present study: optimize preoperative preparation to avoid delays, especially for patients on anticoagulants, performance of stable osteosynthetic/ endoprosthetic reconstructions that allow full weight bearing and promote early postoperative mobilization, as they all are crucial determinants in reducing mortality.

## Data Availability

The data that support the findings of this study are not openly available due to reasons of sensitivity and are available from the corresponding author upon reasonable request. Data are located in controlled access data storage at the UniversityCenter for Orthopedics, Trauma and Plastic Surgery, University Hospital Dresden.
